# Molecular Cloning and Functional Identification of a Pericarp- and Testa-Abundant Gene’s (*AhN8DT-2*) Promoter from *Arachis hypogaea*

**DOI:** 10.3390/ijms25147671

**Published:** 2024-07-12

**Authors:** Yasir Sharif, Yuhui Zhuang, Wenpin Xie, Chong Zhang, Kun Chen, Ye Deng, Yuting Chen, Huiwen Fu, Lihui Wang, Xiangyu Chen, Weijian Zhuang, Hua Chen

**Affiliations:** 1 College of Agriculture, Center of Legume Plant Genetics and System Biology, Institute of Oil Crops, Fujian Agriculture and Forestry University (FAFU), Fuzhou 350002, China; yasirsharif3336@gmail.com (Y.S.); 000q821035@fafu.edu.cn (Y.Z.); wenping777@foxmail.com (W.X.); zhangchong20022008@163.com (C.Z.); kunchen9308@163.com (K.C.); dengye116@163.com (Y.D.); 18094012273@163.com (Y.C.); fuhuiwen0702@163.com (H.F.); wanglihui3080@163.com (L.W.); wellcxy@126.com (X.C.); 2 College of Life Science, Fujian Agriculture and Forestry University, Fuzhou 350002, China; 3 College of Plant Protection, Fujian Agriculture and Forestry University, Fuzhou 350002, China; 4 Crops Research Institute, Fujian Academy of Agricultural Science, Fuzhou 350013, China

**Keywords:** abiotic stress, aflatoxins, constitutive promoter, genome-wide, tissue-specific promoter

## Abstract

Cultivated peanut (*Arachis hypogaea* L.) is a key oil- and protein-providing legume crop of the world. It is full of nutrients, and its nutrient profile is comparable to that of other nuts. Peanut is a unique plant as it showcases a pegging phenomenon, producing flowers above ground, and after fertilization, the developing peg enters the soil and produces seeds underground. This geocarpic nature of peanut exposes its seeds to soil pathogens. Peanut seeds are protected by an inedible pericarp and testa. The pericarp- and testa-specific promoters can be effectively used to improve the seed defense. We identified a pericarp- and testa-abundant expression gene (*AhN8DT-2*) from available transcriptome expression data, whose tissue-specific expression was further confirmed by the qRT-PCR. The 1827bp promoter sequence was used to construct the expression vector using the pMDC164 vector for further analysis. Quantitative expression of the *GUS* gene in transgenic *Arabidopsis* plants showed its high expression in the pericarp. GUS staining showed a deep blue color in the pericarp and testa. Cryostat sectioning of stained *Arabidopsis* seeds showed that expression is only limited to seed coat (testa), and staining was not present in cotyledons and embryos. GUS staining was not detected in any other tissues, including seedlings, leaves, stems, and roots, except for some staining in flowers. Under different phytohormones, this promoter did not show an increase in expression level. These results indicated that the *AhN8DT-2* promoter drives GUS gene expression in a pericarp- and testa-specific manner. The identified promoter can be utilized to drive disease resistance genes, specifically in the pericarp and testa, enhancing peanut seed defense against soil-borne pathogens. This approach has broader implications for improving the resilience of peanut crops and other legumes, contributing to sustainable agricultural practices and food security.

## 1. Introduction

Peanut (*Arachis hypogaea*) seed coat or testa is the outer covering of edible seeds that protects the seeds from environmental damage [1]. Peanut seeds are the most desirable product of the plant and have both economic and nutritional value. The testa contains low concentrations of fats, salts, and proteins (16–18%) [2]. Although testa is typically considered a non-edible part of the peanut seeds, it plays a crucial role in defense responses. Similarly, the outer hard covering of the seed, known as the pericarp, is also an inedible part of the seed and provides mechanical strength against harsh conditions. The pericarp and testa protect the seeds from biotic and abiotic stresses and mechanical damage [3]. Various fungal diseases, including those caused by *Aspergillus* species (aflatoxins), primarily target the edible seeds and deteriorate seed quality [4]. The pericarp and testa are the main physical barriers against aflatoxin invasion [5]. Under disease-favoring conditions, fungal spores invade the seeds, crossing the pericarp and testa barriers to attack the edible seeds. Pericarp and testa with good defensive capacity are important in protecting the seeds. Aflatoxin-resistant genes in these tissues can improve their defensive ability. Pericarp and testa abundant promoters can drive a gene in a pericarp- and testa-abundant manner. Transformation of disease resistance genes under the control of pericarp and testa abundant promoters is a potential solution in protecting peanut seeds from different fungal and bacterial diseases.

Previously, we identified two novel pericarp-specific genes (*AhGLP17-1* and *AhAPY2-1*) from available expression datasets and characterized the expression of their promoters in transgenic *Arabidopsis* plants [6,7]. Promoters of these genes were used to drive the *GUS* gene in *Arabidopsis* plants. Transgenic *Arabidopsis* plants exhibited significant *GUS* gene expression, specifically in pericarp tissues, with no detectable expression in other tissues. However, to the best of our knowledge, not a single study reports the functions of the testa-abundant gene/promoter in peanut. This study aims to fill this research gap by identifying and characterizing a promoter abundantly expressed in the pericarp and testa of peanuts. We identified a pericarp- and testa-abundant promoter from peanut and characterized its expression in transgenic *Arabidopsis*. This promoter was selected from the transcriptome expression in different peanut tissues available at (http://peanutgr.fafu.edu.cn/Transcriptome.php (accessed on 13 April 2019)). The gene Naringenin 8-dimethylallyltransferase 2, chloroplastic (*N8DT-2*) showed increased expression in the pericarp and testa compared to other tissues. *N8DT-2* is an enzyme belonging to the transferase family, particularly transferring alkyl or aryl groups [8]. Genes containing the *UbiA* domain are ubiquitously present across a wide range of organisms, including animals, plants, bacteria, and fungi. These genes generally function as prenyltransferases, particularly aromatic prenyltransferases, facilitating the transfer of isoprenyl groups to aromatic acceptor molecules. This process results in the formation of carbon–carbon (C–C) bonds between the donor and acceptor molecules [9]. In plants, naringenin 8- dimethylallyltransferase was first isolated by [10] from cultured *Sophora flavescens* cells. The cDNA clone provided prenyltransferase activity with naringenin and dimethylallyl diphosphate (DMAPP) as a substrate. This enzyme was named SfN8DT-1 as it was specific for flavanone as prenyl accepter and DMAPP as prenyl donor, and it causes prenylation of the Naringenin substrate at the 8th position [11].

This promoter was selected based on its high expression in the pericarp and testa and minimal or no expression in the embryo. Gene “Naringenin 8-dimethylallyltransferase 2, chloroplastic” *AhN8DT-2* is present on chromosome 11 (B-subgenome) of peanut and possesses a coding sequence (CDS) length of 1164 bp. The 1827 bp upstream sequence of the *AhN8DT-2* gene was used for promoter cloning. Online databases analyzed *cis*-regulatory elements of the *AhN8DT-2* promoter, and its expression in different peanut tissues was confirmed by qRT-PCR. The basic purpose of analyzing this promoter is to explore the resources for fungal diseases resistant breeding. Functional analysis of *AhN8DT-2* promoter in transgenic *Arabidopsis* plants confirmed pericarp- and testa-specific expression without any notable expression in other tissues. This study explores the significance of this promoter in driving a gene in a pericarp- and testa-abundant manner. Successful transformation of stress-responsive genes (especially aflatoxins responsive) under the control of the *AhN8DT-2* promoter will help achieve sustainable peanut production.

## 2. Results

### 2.1. Identification and Analysis of Pericarp- and Testa-Abundant Gene

**Figure 1:**
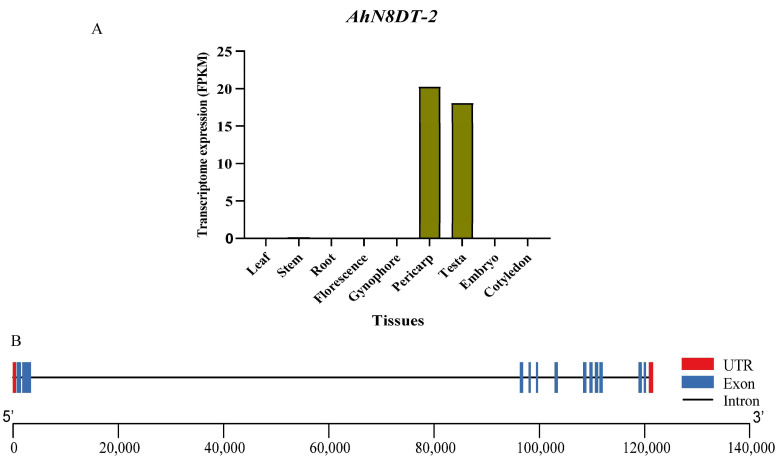
Expression and gene structure of *AhN8DT-2* gene. (**A**) Transcriptome expression of *AhN8DT-2* gene in different peanut tissues. Different tissues are given on the X-axis, while the Y-axis represents their Fragments Per Kilobase of transcript per Million mapped reads (FPKM) values. This graph uses the Mean FPKM values of the embryo, testa, and pericarp. (**B**) Gene structure (exon-intron distribution pattern) of the *AhN8DT-2* gene. Red bars indicate the UTRs (untranslated regions), blue bars indicate the exons, and black lines indicate the introns.

For the identification of pod- and testa-specific expression gene(s), we searched the transcriptomes and microarray expression profiles of the whole peanut genome available in the Peanut Genome Resource (PGR) database [11]. As cotyledons and embryos are the main edible parts of peanut seed, we focused on finding a gene with minimum or no expression in these tissues. From the transcriptome expression datasets, we found a gene belonging to the peanut UbiA prenyltransferase family, “Naringenin 8-dimethylallyltransferase 2, chloroplastic” (*AhN8DT-2*), which showed specifically high expression in the pericarp and testa compared to any other tissue (Figure 1). The *AhN8DT-2* gene with gene ID AH11G07230.1 is present on chromosome 11 of the B sub-genome at 10991400-11112817 position on the positive strand. The *AhN8DT-2* gene is 121,418 bp log and is interrupted by 11 introns of varying lengths, while the second intron is the longest, with 95,798 base pairs, and the ninth intron is the smallest, with 75 base pairs [11]. The 1164bp ORF encodes a protein of 387aa. Protein has a molecular weight of 43.83 kDa and a theoretical isoelectric point of 9.87. The transcriptome expression data showed that the *AhN8DT-2* gene is highly expressed in the pericarp and testa (Table S1); however, microarray expression showed its high expression in the testa only (Table S2). The CDS, protein, and promoter sequence of the *AhN8DT-2* gene are given in File S1.

### 2.2. Expression Validation of AhN8DT-2 Gene in Different Tissues

**Figure 2:**
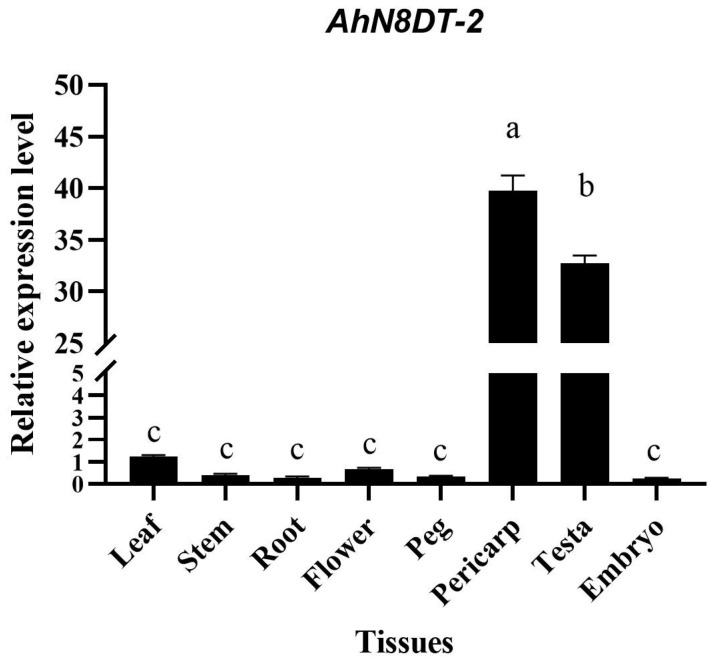
Expression validation of *AhN8DT-2* gene in different tissues of peanut by qRT-PCR. *AhN8DT-2* showed high expression in the pericarp and testa, while the expression level was too low in all other tissues. Data were analyzed by 2^−ΔCT^ method, and significance levels were determined by ANOVA. Letters a, b, and c represent the significance levels with α = 0.05.

It was evident from the transcriptome and microarray expression datasets that the *AhN8DT-2* gene was specifically expressed in pod and seed testa. This specific nature of expression was further verified by qRT-PCR analysis in different tissues. qRT-PCR was performed with specifically designed primers for the *AhN8DT-2* gene, keeping peanut *Actin* as a housekeeping gene. Relative expression in different tissues was calculated by the 2^−ΔCT^ method. The qRT-PCR analysis results indicated a high expression level of the *AhN8DT-2* gene in pericarp and testa samples. In contrast, expression in other tissues, including leaf, stem, root, flower, and peg, was very low. Specifically, the expression was very low in the embryo (Figure 2). Quantitative PCR results correspond to the transcriptome expression profiles of the *AhN8DT-2* gene. Statistical analysis was performed using one-way ANOVA, and the differences in expression levels among different tissues were found to be statistically significant (*p* < 0.05). Post hoc comparisons using the LSD test indicated that the expression levels in the pericarp and testa were significantly higher than in other tissues (*p* < 0.05). The qRT-PCR-based validation suggested that the promoter of the *AhN8DT-2* gene can be used as a pericarp- and testa-abundant promoter to drive a gene in pericarp and testa only.

### 2.3. In Silico Analysis of AhN8DT-2 Promoter

**Figure 3:**
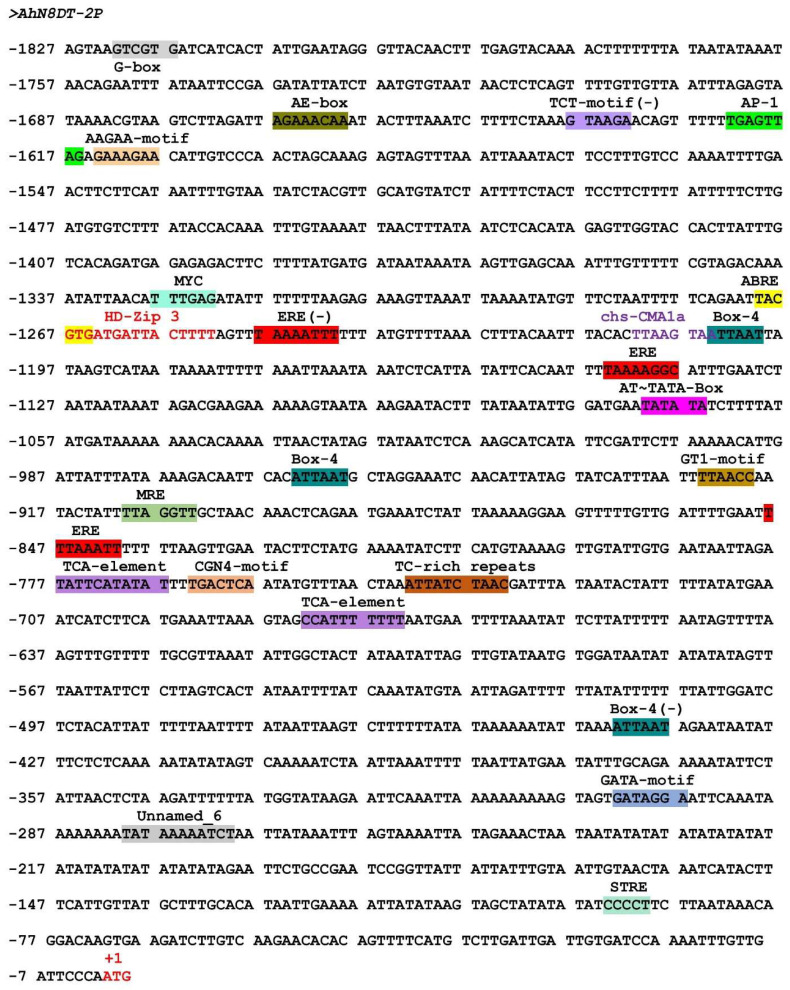
Prediction of *cis*-regulatory elements in *AhN8DT-2* promoter by the PlantCARE database. Online prediction identified several key elements needed for promoter functioning.

The 1827bp promoter sequence was submitted to online promoter analysis databases, PlantCARE, and PLACE databases to analyze upstream cis-regulatory elements. These online prediction databases showed that the *AhN8DT-2* promoter has core promoter elements, including the TATA box and CAAT box, required for accurate transcription starting and tissue-specific activity, respectively [12,13]. Several other key regulatory factors were also predicted, such as elements responsive to light induction (AE-box, Box 4, G-Box, GATA-motif, and GT1-motif), hormone-related factors ethylene (ERE), gibberellin (TATC-box), salicylic acid (TCA-element), and abscisic acid (ABRE). Further, elements related to defense responses (MYB binding sites and TC-rich repeats), anaerobic induction-related elements (ARE), and wound-responsive factors (WUN-motif) were also present. Detailed information on cis-elements in *AhN8DT-2P* is shown in Figure 3 and Table S3.

Prediction of transcription factors (TFs) binding sites and *cis*-elements with the PLACE database also provided the presence of several key TF binding sites and regulatory elements, such as seed-specific elements (RY-repeats) [14], binding sites for MYB, WRKY, AP2, etc. These elements and binding sites strongly suggest that the *AhN8DT-2* promoter could be an appropriate candidate for a gene’s native promoter. Aside from these previously reported *cis*-elements, an unknown element was also predicted in the *AhN8DT-2* promoter (Figure 3).

### 2.4. Cloning of AhN8DT-2 Promoter and Generation of Transgenic Arabidopsis Plants

**Figure 4:**
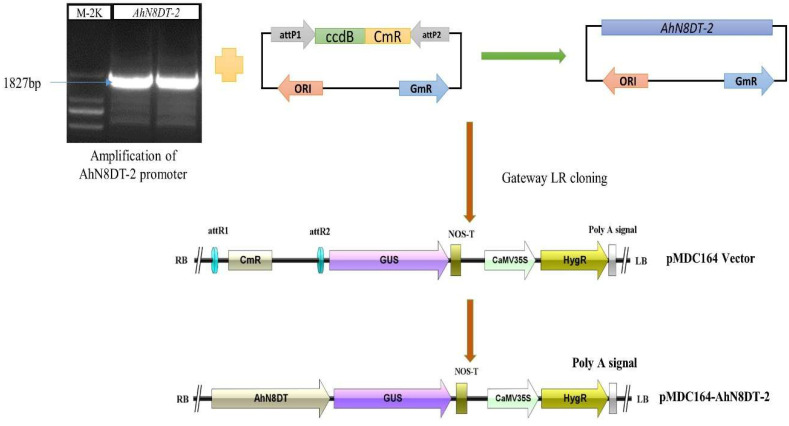
Isolation and vector construction for *AhN8DT-2* promoter. An 1827 bp region was amplified with promoter-specific primers and cloned into entry vector (pDONR207) by Gateway BP reaction. After sequence confirmation, the promoter fragment was cloned into the plant expression vector (pMDC164) by Gateway LR reaction.

For cloning of the *AhN8DT-2* promoter, the 1827bp upstream region was PCR-amplified from the DNA template peanut variety XHXL using specific primers (Table S5). After verifying the promoter sequence, the gateway adapter sequence was annealed to the promoter fragment by PCR with gateway primers. Then, the entry vector was constructed by annealing the promoter between the attP sites of the pDONR207 vector through BP reaction. The promoter sequence was verified again and then annealed between the attR sites of the pMDC164 expression vector through LR-cloning. In this way, the expression vector pMDC164-AhN8DT-2P was successfully constructed. The complete process of vector construction is shown in Figure 4. The vector was transformed into *A. tumefaciens* cells by the method of heat shock. Expression vector harboring *Agrobacterium* colonies selected on YEB + Rif + Km selection medium and further used for genetic transformation of *Arabidopsis* plants by floral dipping [15]. Positive T0 plants were selected on MS+HygR medium and genetically verified through PCR with genomic DNA. The positively transformed seedlings were healthy and green, while the non-transformed seedlings turned yellow and stopped growing. Eight healthy and green seedlings were verified by PCR-based amplification.

### 2.5. Molecular Analysis of Transgenic Arabidopsis and Generation of Pure Lines

**Figure 5:**
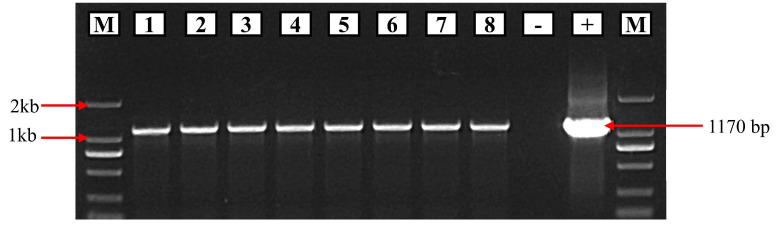
Genetic validation of T0 generation of transgenic *A. thaliana* plants. Eight Hygromycin-resilient plants were confirmed by PCR amplification. +ve control = plasmid of expression vector, −ev control = DNA from Col-0 M = 2 kb marker.

For the confirmation of successfully transplanted *Arabidopsis thaliana* plants, DNA was extracted from the leaf samples of transgenic plants resistant to hygromycin using the CTAB reagent. Promoter-based forward and GUS gene-based reverse primers were used for PCR confirmation. The plasmid of the expression vector (LR-construct) was used as the positive control, while the negative control consisted of the DNA from non-transformed Col-0 plants (Figure 5). Eight PCR-confirmed plants were selected and grown to T3 homozygous generation with hygromycin resistance and PCR amplification screening was performed in each generation. Great care was taken in each generation to avoid outcrossing.

### 2.6. Tissue-Specific Induction of AhN8DT-2 Promoter

**Figure 6:**
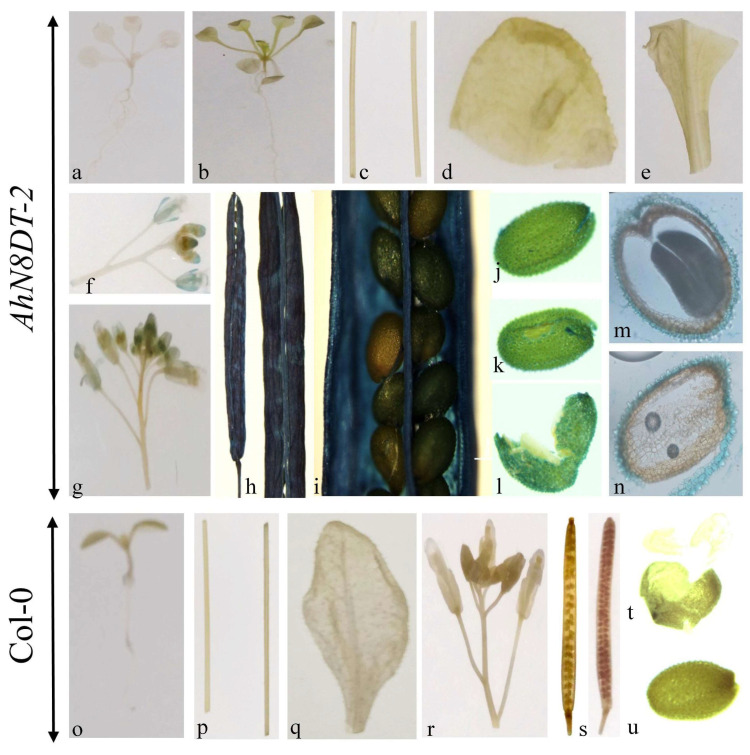
GUS staining of different tissues/organs of transgenic (**a**–**n**) and non-transgenic (**o**–**u**) *A. thaliana* plants. (**a**,**b**) = seedlings, (**c**) = stems, (**d**,**e**) = leaves, (**f**,**g**) = flowers, (**h**,**i**) = siliques, (**j**,**k**) = seeds, (**l**) = physically ruptured seeds, (**m**,**n**) = seeds cryostat sectioning of transgenic plants, (**o**) = seedling, (**p**) = stem, (**q**) = leaf, (**r**) = flower, (**s**) = siliques, (**t**) = physically ruptured seed, (**u**) = intact seed of non-transgenic plants. GUS staining showed a dense blue color in the pericarp and testa. Flowers also showed mild staining.

For the GUS staining essay, different tissues/organs of transgenic plants were incubated in GUS solution for 12 h at 37 °C. After incubation, the samples were washed and decolorized with 75% ethanol. These tissue samples showed deep staining (blue color) in siliques of transgenic plants. The dark blue color was also present in the seed coat of transgenic seeds. Young seedlings and other vegetative tissues were not stained (Figure 6). Light staining was also detected in flowers. Seeds were ruptured before staining to facilitate the penetration of the staining solution into cotyledons and embryos. Ruptured seeds were proceeded for cryostat sectioning to check the staining in different seed tissues under the microscope, and there was no staining in cotyledons and embryos (Figure 6). The staining results were compared by using *A. thaliana* wild-type Col-0 plants as a control. The staining results demonstrated that *AhN8DT-2P* is abundantly and highly expressed in the pericarp and testa without showing expression in any other tissues. Statistical analysis of the GUS staining intensity was performed using one-way ANOVA, and the differences between the pericarp/testa and other tissues were found to be statistically significant (*p* < 0.05). Overall, the staining results suggest that the *AhN8DT-2* promoter is a strong pericarp- and testa-abundant promoter and the right substitute to express a gene in a pericarp- and testa-specific manner.

### 2.7. Quantification of GUS Gene Expression by qRT-PCR

**Figure 7:**
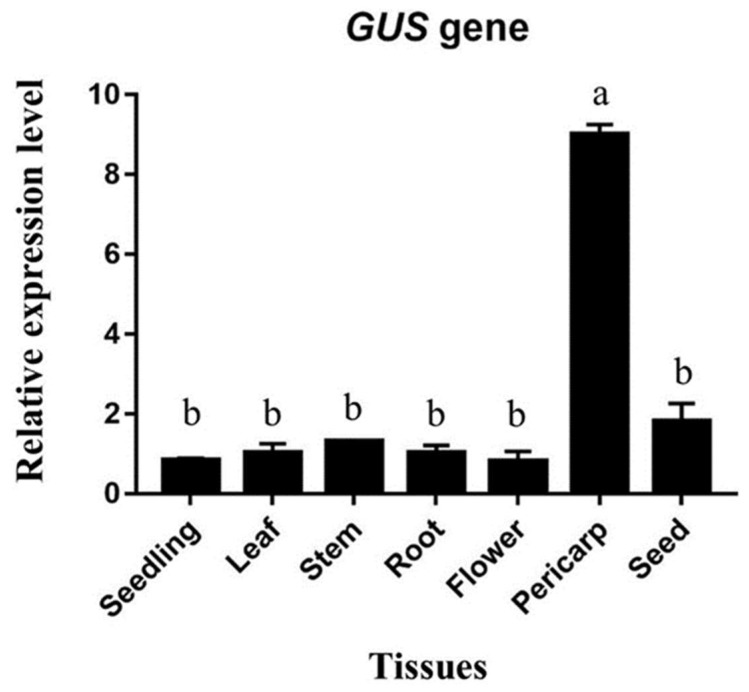
Quantitative expression of *GUS* gene under the control of *AhN8DT-2P* in different tissues of transgenic *Arabidopsis* plants. The *GUS* gene under the control of the *AhN8DT-2* promoter showed comparatively high expression in the pericarp of transgenic *Arabidopsis* plants. Letters a and b show the statistical significance levels with *p* = 0.05.

The real-time expression of the *UidA* gene (GUS) in different tissues of transgenic plants was analyzed by qRT-PCR (Figure 7). For the qRT-PCR analysis, RNA from seedlings, leaves, roots, stems, flowers, pericarp (silique outer coverings), and seeds were extracted using the TriQuick reagent. RNA from whole seeds was extracted to measure the expression in the seed coat, embryo, and cotyledons together. Quantitative real-time PCR revealed a comparatively higher expression of the *GUS* gene in the pericarp of transgenic plants than in any other tissues (Figure 7). 

### 2.8. Expression Responses of AhN8DT-2P to Different Hormonal Treatments

**Figure 8:**
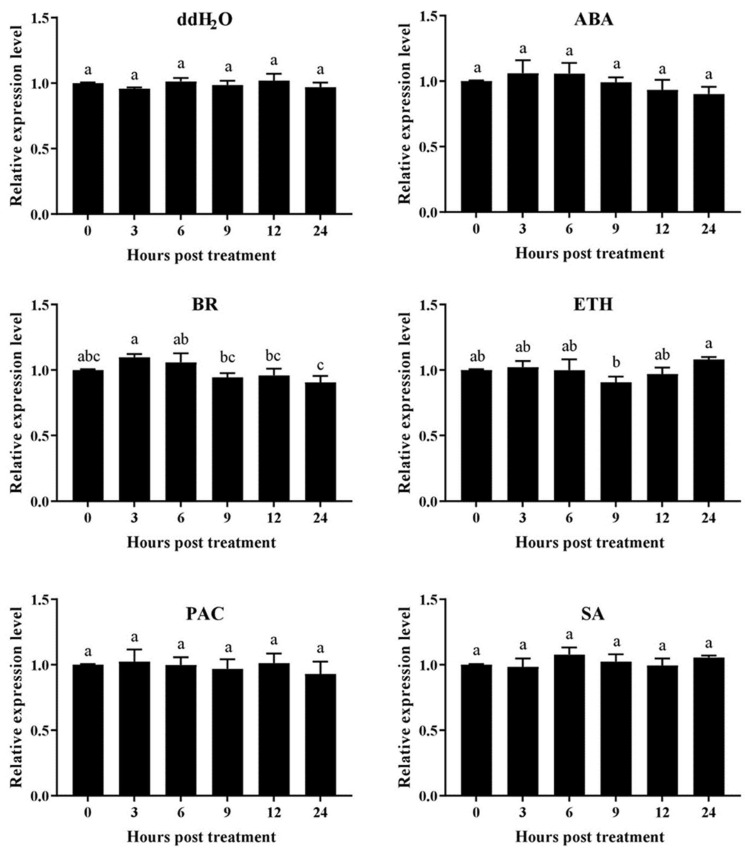
The response of the *AhN8DT-2*-controlled GUS gene to different hormone treatments was that there was no change in the expression level of the GUS gene in response to different hormones and ddH2O spray at all time points (in accordance with transcriptome expression). ABA = abscisic acid, BR = brassinolide, ETH = ethphon, PAC = paclobutrazol, and SA = salicylic acid. Data were analyzed by analysis of variance (ANOVA), letters a, b and c show the statistical significance levels with *p* < 0.05).

The expression level of *AhN8DT-2* promoters in response to different growth regulators was determined by checking the expression of the *GUS* gene in transgenic plants treated with growth regulators. Transgenic *Arabidopsis* plants were sprayed with abscisic acid, brassinolide, ethephon, paclobutrazol, and salicylic acid solutions for that purpose. Plants were also sprayed with distilled water. Under all regulators and distilled water treatments, the *GUS* gene did not show any remarkable change in expression (Figure 8). The expression levels before and after treatment with regulators were almost similar except for some minor deviations, indicating that the *AhN8DT-2* promoter did not cause any change in the expression of the *GUS* gene in response to different regulators and water. In the transcriptome expression dataset of the peanut genome, the *AhN8DT-2* gene did not respond to hormones, growth regulators, and ddH_2_O treatment (Table S6). Quantitative PCR results also confirmed a similar expression trend of *AhN8DT-2* promoter in transgenic plants. A deep GUS staining in the pericarp and testa of transgenic plants compared to all other tissues and expression profiling under different hormones indicated that the *AhN8DT-2* promoter is a strong candidate for pericarp- and testa-specific expression without being expressed by hormone signaling. Overall, these results confirmed that the *AhN8DT-2* promoter could be used to derive the expression of a foreign gene in a pericarp- and testa-specific manner.

## 3. Discussion

During gene expression, promoters play a vital role in transcriptional regulation. The expression sites of a gene can be correctly and reliably located by analyzing the temporal and spatial expression patterns of its promoter, which is crucial for investigating the temporal and spatial expression profiles of genes and establishing gene function [16]. Selecting an appropriate promoter is essential for generating genetically modified (GM) crops [17]. Producing transgenic plants with increased yield without compromising environmental or biosafety issues has significant implications. Improvements in the expression of transgenes have been achieved by using hybrid or combination promoters derived from constitutive promoters. It is often believed that constitutive promoters are advantageous for achieving a high expression level of selectable marker genes, which is required to successfully select transgenes [18]. However, using active constitutive promoters in GM plants is not always ideal since constitutive overexpression might reduce the growth rate by competing for resources such as the energy required for RNA biosynthesis, protein building blocks, and others. To minimize the unintended consequences of harsh environments like heat, cold, drought, and salt, tissue-specific or stress-inducible promoters can be used instead of constitutive promoters [19]. Tissue-specific and inducible promoters are beneficial resources for the genetic engineering of plants because the expression of genes under their control can function at a specific developmental stage or in a particular tissue, resulting in a reduced amount of energy expended by the plant [20]. Furthermore, these promoters will be active only when necessary, allowing yield or productivity to be maintained even under stressed environments.

The identified *AhN8DT-2* promoter has significant potential applications in crop improvement, particularly in enhancing the defense mechanisms of peanut seeds against soil-borne pathogens. By driving the expression of disease-resistance genes, specifically in the pericarp and testa, this promoter can help develop peanut varieties with increased resilience to fungal infections, including aflatoxins. This approach can be extended to other legume crops, contributing to sustainable agricultural practices and food security. As this promoter specifically drives the expression of genes in pericarp/test (non-edible tissues), it will have fewer regulatory approval implications. However, challenges such as the efficiency of gene transformation and the environmental impact of these modifications need to be addressed. Future studies should focus on optimizing transformation protocols and evaluating the long-term stability and effectiveness of transgene expression under field conditions.

Earlier research indicated that the seedless grape *VvβVPE* gene was exclusively expressed in the ovule, and the expression of the *VvβVPE* gene in seeded grapes was enhanced throughout the seed development process. In contrast, the seedless grape *VvβVPE* gene was clearly expressed during seed abortion [21]. In different plant species, *βVPE* showed tissue specificity in different ways. In rice, it showed constitutive expression while being expressed in the leaf sheaths, leaves, stems, panicles, and the nascent endosperm [22], but in *Arabidopsis*, *VPE* is expressed in a seed-specific manner [23,24]. Tang et al. (2016) demonstrated that *βVPE* expressed differently in different organs/tissues of the grapevine. Aside from the seed, the roots had just a mediocre amount of expressiveness. The desired expression of a gene at a certain developmental stage or in a specific tissue is controlled by tissue-specific expression promoters expressed in particular tissues and organs [25].

Peanut is an important grain legume in tropical and subtropical areas that provides oil and proteins. Due to its geocarpic nature, it is affected by different soil-borne pathogens and diseases. Peanut’s outer hard shell (pericarp) and inner thin seed coat (testa) are important defense organs that protect the seeds from different biotic and abiotic stresses and mechanical damage. Any pathogen invasion is associated with the breakdown of these barriers. The defensive ability of these organs depends upon the genetic and molecular mechanisms undergoing in these organs. Disease-responsive genes specifically expressed in these organs can improve plant defense against stress agents. Modern biotechnology techniques are making it easier to generate genetically engineered crops, and a greater number of biofortified crops are becoming available [26,27,28]. Crop biofortification programs are based on introducing new metabolic pathways in seed or endosperm development. A seed or endosperm-specific promoter directs each new pathway. For example, genetically modified *Brassica juncea* has been used to produce DHA (docosapentaenoic acid), where a plasmid containing nine genes under the control of a single seed-specific promoter (napin) was transformed [29]. This example highlights the importance of specific expression promoters in crop genetic engineering.

Similarly, new metabolic pathways can be introduced in peanut pericarp and testa to increase their response to different pathogen attacks. To achieve this goal, pericarp- and testa-abundant/specific promoters are of great importance as they can drive a disease resistance gene in a pericarp- and testa-abundant/specific manner. This was why a pericarp- and testa-abundant gene, without expressing it in other tissues was selected. The gene *AhN8DT-2* was found to be specifically expressed in the pericarp and testa. The qRT-PCR results confirmed the pericarp- and testa-abundant expression of the *AhN8DT-2* gene. Analysis of the promoter’s *cis*-regulatory elements indicated the presence of many important *cis*-regulatory elements responsible for a gene’s expression in response to light, hormones, and other abiotic stresses. The number and type of *cis*-elements determine the strength and specificity of a promoter [30]. The presence of these regulatory elements suggests that this promoter could also be stress-inducible, but it is not necessary that predicted elements should be functionally active in driving expression in an inducible manner [31]. 

The specificity of the *AhN8DT-2* promoter activity in pericarp and testa tissues can be attributed to unique *cis*-regulatory elements and transcription factor binding sites identified in the promoter region. These elements likely interact with tissue-specific transcription factors that are abundantly expressed in the pericarp and testa, leading to the selective activation of the promoter in these tissues. Further studies are required to identify and characterize these transcription factors and their interaction with the *AhN8DT-2* promoter to gain a deeper understanding of the regulatory mechanisms involved. While this study provides valuable insights into the *AhN8DT-2* promoter, it has some limitations. The functional validation of the promoter was conducted in *A. thaliana*, and further validation in peanut plants is necessary to confirm its effectiveness in the native species. Additionally, the long-term stability and potential off-target effects of the promoter-driven transgene expression must be thoroughly investigated. Future research should also explore the interaction of the *AhN8DT-2* promoter with various biotic and abiotic stress conditions to understand its broader applicability.

Future research should focus on validating the effectiveness of the *AhN8DT-2* promoter in peanut plants under various environmental conditions. This includes developing transgenic peanut lines expressing disease resistance genes driven by the *AhN8DT-2* promoter and evaluating their performance in field trials. Additionally, exploring the potential of the *AhN8DT-2* promoter to drive the expression of other beneficial genes, such as those involved in abiotic stress tolerance and nutrient uptake, could further enhance crop resilience. Collaborative efforts between molecular biologists, plant breeders, and agronomists will be essential to translate these findings into practical applications in peanut breeding programs.

The genetic transformation of peanut is still a major bottleneck for the functional investigation of promoters and genes; that is why *Arabidopsis* has become an ideal plant for characterizing promoters and genes. We chose *Arabidopsis* to characterize the functional expression of the *AhN8DT-2* promoter. Transgenic *Arabidopsis* plants showed that the *AhN8DT-2* promoter drove the expression of the *GUS* gene in a pericarp- and testa-abundant manner, as deep staining was present in these tissues of transgenic *Arabidopsis* seeds. Quantitative expression of the *GUS* gene also showed a high expression level in pericarp tissues. However, some staining was also present in flowers, possibly due to species variation. These results indicate that the *AhN8DT-2* promoter can drive gene expression in a pericarp- and testa-abundant manner. Previously, we identified and functionally characterized two pericarp-specific promoters, *AhGLP17-1P* [7] and *AhAPY2-1p* [6], from peanut. Functional studies of these promoters also supported the results of *AhN8DT-2P*. The novel thing about *AhN8DT-2P* is that it is specifically expressed in both pericarp and testa, while others are expressed in the pericarp only. Using this promoter to modify the genetic architecture of the pericarp and testa of peanut is a good option to increase peanut resistance against soil and seed-borne diseases and pathogens. This goal could be achieved by successfully transforming disease resistance genes under the control of *AhN8DT-2* promoter in peanut. It would be a groundbreaking achievement in peanut breeding. 

## 4. Materials and Methods

### 4.1. Plant Materials and Growth Conditions

*Arabidopsis* (Col-0) and peanut cultivar Xinhuixiaoli (XHXL) were used in this study. Seeds of *Arabidopsis* and peanut were maintained by the Center for Legume Plants Genetics and Systems Biology, Fujian Agriculture and Forestry University (FAFU). Peanut seeds were grown in the greenhouse and research fields of FAFU in Sanming County, Fujian province, while *Arabidopsis* plants were grown in the greenhouse. The greenhouse temperature was maintained at 25 °C, and a day/night photoperiod cycle of 16/8 h was maintained. The relative humidity level of the greenhouse was maintained at 75%.

### 4.2. Identification of Pericarp- and Testa-Abundant Expression Gene

Pericarp- and testa-abundant expression genes were identified by analyzing the transcriptome and microarray expression data of the whole peanut genome, available at the Peanut Genome Resource (PGR) database (http://peanutgr.fafu.edu.cn/ (accessed on 13 April 2019)) [11]. Through genome-wide mining of the PGR database, a gene belonging to the *UbiA* family, “Naringenin 8-dimethylallyltransferase 2, chloroplastic” (*AhN8DT-2*) with the PGR ID AH11G07230.1, was found to be specifically expressed in the pericarp and testa. We selected this gene for further analysis and promoter cloning.

### 4.3. Bioinformatics Analysis of AhN8DT-2 Gene and Promoter

The basic information of the *AhN8DT-2* gene was obtained from the PGR database, while different physicochemical properties were identified from the ExPASy server (https://web.expasy.org/protparam/ (accessed on 21 April 2019)) [32]. To predict the expression characteristics of the *AhN8DT-2* promoter, the 1827 bp upstream region of the *AhN8DT-2* gene was used to identify different cis-elements and transcription factors binding sites. Different cis-elements were identified through the PlantCARE (https://www.dna.affrc.go.jp/PLACE/?action=newplace (accessed on 21 April 2019)) [33] and the PLACE (http://bioinformatics.psb.ugent.be/webtools/plantcare/html/ (accessed on 21 April 2019)) [34] databases. 

### 4.4. Expression Validation of AhN8DT-2 Gene in Various Tissues

The expression of the *AhN8DT-2* gene in different tissues was analyzed using qRT-PCR. Samples were taken from peanut plants grown at the Sanming research station. Various tissue/organ samples, including root, stem, young leaves, flower, gynophore (peg), shell (pericarp), testa, and embryo, were collected and stored in liquid nitrogen. The collected samples were preserved at −80 °C before RNA extraction. Total RNA was extracted from the collected tissues using the CTAB method [35] with some modifications. Primers for the qRT-PCR analysis were designed with greater care using the Primer Primier 5.0 software. Primers were blasted in NCBI and PGR databases to check their uniqueness. Primer sequences are given in Table S5. The qRT-PCR reactions were conducted on an ABI 7500 system using the Taq Pro Universal SYBR qPCR Master Mix (Vazyme Biotech Co., Ltd., Nanjing, China) under the following cycling conditions: 94 °C (1 min), 60 °C (1 min), and 72 °C (1 min) for 40 cycles. The peanut *Actin* gene was used as an internal control. 

### 4.5. DNA Isolation and Promoter Amplification

The *AhN8DT*-2 promoter was cloned from the genomic DNA of the XHXL peanut variety. For DNA isolation, leaf samples were collected from XHXL plants grown in the greenhouse at the 4–6 leaf stage. DNA was extracted using the CTAB (2% CTAB, 0.5 M Na-EDTA, 1 M Tris-HCl, 1.4 M NaCl, 3% PVP-40, and 0.2% β-Mercaptoethanol) method [36] with few changes. An 1827bp 5′ flanking region of the *AhN8DT-2* gene was amplified with specific primers using the PrimeSTAR^®^ Max DNA polymerase (Takara, Dalian, China). The PCR product was purified with the TIANGEN Universal DNA Purification Kit (Tiangen Biotech, Beijing, China). The amplified promoter region was cloned into the pMD19T vector and sequenced by the Beijing Genomics Institute (BGI, Shenzhen, China). Specifically designed primers were used to amplify the promoter region (Table S5). 

### 4.6. Cloning of AhN8DT-2 Promoter into pMDC164 Vector

After verifying the *AhN8DT-2* promoter sequence, the next step was to construct the plant expression vector. The expression vector was constructed using a two-step gateway cloning. First, the promoter fragment was amplified with primers containing gateway universal adapters. Then, the promoter fragment was ligated into the pDONR207 vector using the BP Clonase enzyme (ThermoFisher Scientific, Waltham, MA, USA) with BP reaction. At this stage, the promoter sequence was confirmed once again, and after sequence verification, it was cloned into the pMDC164 vector using the LR Clonase enzyme (ThermoFisher Scientific, USA) with LR cloning. The successfully constructed vector was then mobilized to *Agrobacterium tumefaciens* (GV3101) using the heat shock method. 

### 4.7. Transformation into Arabidopsis

*Agrobacterium* cultures containing the pMDC164-*AhN8DT-2P* construct were grown to the logarithmic growth stage, with an OD600 of 1.0–1.5 by incubating them at 28 °C, 220 rpm in YEB/Km/Rif medium (Km = 50 µg mL^−1^, Rif = 75 µg mL^−1^). The *Agrobacterium* cells were harvested by centrifugation for 10 min at 4000 rpm and then resuspended in a solution of sucrose (5% sucrose, 100 µg mL^−1^ Acetosyringone (AS), and 0.02% Silwet L-77) [37]. The bacterial cells with the sucrose solution were kept at 4 °C for two hours before the transformation. *Arabidopsis* plants were genetically transformed with the vector constructs using the floral dip method [15]. Unopened flowers were dipped in the vector-containing solution for 10–15 s and then kept in the dark for 24 h. Opened flowers and siliques were removed before the transformation, and after seven days, the transformation was repeated. After the transformation, plants were grown normally in the greenhouse, and T0 seeds were harvested.

### 4.8. Screening of Positive Plants and Generation of Pure Lines

About 500 T0 seeds were vernalized at 4 °C for 48 h and then sterilized with 75% ethanol and 10% H_2_O_2._ The surface-sterilized seeds were spread over the solid MS medium with 50 µg mL^−1^ hygromycin antibiotic. Seedlings were observed after one week of germination. Healthy and green seedlings (resistant to hygromycin) were transferred to 6 cm diameter pots. Eight plants were confirmed by PCR with promoter-specific forward and *GUS* gene-specific reverse primers. After genetic verification through PCR, the seedlings were grown to maturity, and great care was taken to avoid outcrossing. In this way, T3 homozygous generation was obtained, while antibiotic resistance and genetic verification by PCR were performed in each generation.

### 4.9. Histochemical GUS Expression

Different tissues of T3 *Arabidopsis* plants, including leaves, stems, flowers, roots, siliques, seeds, and young seedlings, were taken for the GUS staining experiment [38]. Samples were placed in GUS solution, 2 mM 5-Bromo-4-chloro-3-indolyl β-D-glucuronide (X-Gluc), with 50 mM sodium phosphate buffer, 10 mM EDTA, 2 mM potassium ferricyanide, and potassium ferrocyanide (each), 0.1% Triton X-100, and incubated for 12 h at 37 °C. After 12 h of incubation, chlorophyll was destained with 75% ethanol. Samples were photographed with a digital camera and an Olympus microscope (BX3-CBH) (Olympus Corporation, Tokyo, Japan).

### 4.10. Real-Time Expression of UidA Gene

The real-time expression of the *UidA* gene (*GUS*) in various tissues of transgenic plants was observed by qRT-PCR. Total RNA was extracted by TRIzol (Life Technologies, Carlsbad, CA, USA), reagent, and 1.5 µg RNA was converted to cDNA by PrimeScript 1st strand cDNA Synthesis Kit (Takara, Dalian, China). The ABI 7500 instrument was used for qRT-PCR reaction with the aforementioned conditions. The *Arabidopsis Actin* gene was used as the housekeeping gene to normalize the expression level. 

### 4.11. Cryostat Sectioning Analysis of Transgenic Arabidopsis Seeds

Cryostat sectioning of T3 seeds was performed to check the histochemical expression of the *UidA* gene in cotyledons, testa, and embryos. Sectioning was performed using the Leica CM1950 Cryostat Microtome (Leica Biosystems, Wetzlar, Germany). Mechanically ruptured seeds of the T3 generation were incubated in GUS solution so that the staining solution could penetrate the seed tissues. After overnight incubation at 37 °C, the stained seeds were washed and decolorized with 75% ethanol. The cryostat microtome was turned on three hours before use to maintain a temperature of −20 °C. The required materials, including brushes, specimen discs, forceps, etc., were put inside the cooling chamber. Sections of 50 µm thickness were made and photographed by an Olympus microscope (IX73) (Olympus Corporation, Tokyo, Japan).

### 4.12. Evaluation of AhN8DT-2P under Different Phytohormones

Transgenic *Arabidopsis* plants harboring *AhN8DT-2P* were treated with different phytohormones/growth regulators to determine whether *AhN8DT-2* promoter expression corresponds to transcriptome expression profiling. These hormones/regulators included abscisic acid (ABA, 10 μg/mL), brassinolide (BR, 0.1 mg/L), ethephon (ETH, 1 mg/mL), salicylic acid (SA, 3 mmol/L), paclobutrazol (PAC, 150 mg/L), and distilled water. Young transgenic plants were sprayed with these hormones/regulators. Samples for RNA extraction were taken before treatment (0 h or control), and after 3 h, 6 h, 9 h, 12 h, and 24 h of hormone spray. As mentioned above, RNA was extracted using TriQuick reagent, and 1.5 μg RNA was converted into cDNA. The quantitative expression of the *UidA* gene (*GUS*) under the *AhN8DT-2* promoter was assessed by qRT-PCR.

### 4.13. Statistical Analysis

The data for qRT-PCR were analyzed by the 2^−ΔCT^ method. One-way ANOVA was used to determine the statistical significance of the expression levels among different tissues and under different hormone treatments. Means were compared by LSD test at a 5% significance level.

## 5. Conclusions

Modern biotechnological techniques provide robust tools to change the genetic architecture of crop plants by incorporating new metabolic pathways. These techniques also provide the facilities to investigate and characterize the genes and promoters for their possible uses in plant biofortification programs. This study investigated the expression pattern of a pericarp- and testa-abundant promoter from peanut in transgenic *Arabidopsis*. The *AhN8DT-2* promoter was selected based on its pericarp- and testa-specific expression from the available microarray and transcriptome expression data. It showed strong pericarp- and testa-abundant expression in transgenic *Arabidopsis* plants. To the best of our knowledge, no information was previously available on the pericarp- and testa-abundant expression promoter in peanut. This promoter will provide a base for the future breeding programs of peanut aimed at changing the genetic architecture of non-edible seed tissues, especially for driving resistance-related genes in a pericarp- and testa-abundant manner. 

## Data Availability

The datasets presented in this study can be found in the online PGR database http://peanutgr.fafu.edu.cn/ (accessed on 13 April 2019). Moreover, the datasets used and/or analyzed during the current study are available from the corresponding author upon reasonable request. However, most of the data are shown in the Supplementary Files.

## Supplementary Materials

The following supporting information can be downloaded at: https://www.mdpi.com/article/10.3390/ijms25147671/s1.
