# Supplementary material for: Molecular Cloning and Functional Identification of a Pericarp- and Testa-Abundant Gene’s (AhN8DT-2) Promoter from Arachis hypogaea
Source: Int J Mol Sci. 2024 Jul 12;25(14):7671. doi: 10.3390/ijms25147671 (PMC11276643; doi:10.3390/ijms25147671)
Supplement: Supplementary file 1 [file ijms-25-07671-s001.zip › Table S1.pdf]

Table S1. Transcriptome expression of *AhN8DT-2* gene in different tissues of peanut.

Tissue	FPKM
Leaf	0
Stem	0.2
Root	0
Florescence	0
Gynophore	0.08
Pericarp-I	0
Pericarp-II	2.66
Pericarp-III	58.19
Testa -I	1.21
Testa -II	34.93
Embryo-I	0
Embryo -II	0
Embryo -III	0
Embryo-IV	0
Cotyledon	0

Note;

Pericarp-1= pericarp samples 10-20 days after pegging

Pericarp-II= pericarp samples 30-40 days after pegging

Pericarp-III= pericarp samples 50-60 days after pegging

Embryo-I= Embryo samples 10-20 days after pegging

Embryo-II= Embryo samples 20-30 days after pegging

Embryo-III= Embryo samples 30-40 days after pegging

Embryo-IV= Embryo samples 50-60 days after pegging

Testa-I= Testa samples 30 days after pegging

Testa-II= Testa samples 50 days after pegging
