# Supplementary material for: Molecular Cloning and Functional Identification of a Pericarp- and Testa-Abundant Gene’s (AhN8DT-2) Promoter from Arachis hypogaea
Source: Int J Mol Sci. 2024 Jul 12;25(14):7671. doi: 10.3390/ijms25147671 (PMC11276643; doi:10.3390/ijms25147671)
Supplement: Supplementary file 1 [file ijms-25-07671-s001.zip › Table S2.pdf]

Table S2. Microarray expression of *AhN8DT-2* gene in different tissues of peanut.

Embryo-I	52.81
Embryo -II	105.79
Embryo -III	216.3
Embryo-IV	152.98
Pericarp-I	152.77
Pericarp-II	185.03
Pericarp-III	375.16
Testa	1115.96
Leaf	168.13
Root	161.35
Stem	280.7
Florescence	143.46
Gynophore	243.25

Note;

Pericarp-1= pericarp samples 10-20 days after pegging

Pericarp-II= pericarp samples 30-40 days after pegging

Pericarp-III= pericarp samples 50-60 days after pegging

Embryo-I= Embryo samples 20 days after pegging

Embryo-II= Embryo samples 30-40 days after pegging

Embryo-III= Embryo samples 50-60 days after pegging

Testa samples of 20 days, 30-40 days, and 50-60 days were mixed for microarray expression analysis.
