# Supplementary material for: Molecular Cloning and Functional Identification of a Pericarp- and Testa-Abundant Gene’s (AhN8DT-2) Promoter from Arachis hypogaea
Source: Int J Mol Sci. 2024 Jul 12;25(14):7671. doi: 10.3390/ijms25147671 (PMC11276643; doi:10.3390/ijms25147671)
Supplement: Supplementary file 1 [file ijms-25-07671-s001.zip › Table S5.pdf]

**Table S5.** Primers used to study the *AhN8DT-2* promoter

| Primer Name | Primer Sequence |
|---|---|
| **qRT-PCR** | |
| AhActin-F | GAGGAGAATCAGAAGCAAGTC |
| AhActin-R | CATATACAGCATAGCGGCACTC |
| q-AhN8DT-2F | CAATCCTTGGAGCATAACTGG |
| q-AhN8DT-2R | GATGTCGAAGATATGCTCATTA |
| **Promoter amplification** | |
| AhN8DT-2F | AGTAAGTCGTGATCATCACTATTG |
| AhN8DT-2R | GGAAGTGGAAGCCATTGGGAATC |
| Promoter Gateway cloning | |
| gwAhN8DT-2F | GGGGACAAGTTTGTACAAAAAAGCAGGCTTCAGTAAGTCGTGAT CATCACTATTG |
| gwAhN8DT-2R | GGGGACCACTTTGTACAAGAAAGCTGGGTCGGAAGTGGAAGCCA TTGGGAATC |
| **Verification of transgenic Arabidopsis plants** | |
| Pro-GUS-F | GGTTGCTAACAAACTCAGAATGAAATC |
| Pro-GUS-R | ACTGATCGTTAAAACTGCCTGGCA |
| **Quantitative expression of *GUS* gene** | |
| q-GUS-F | AGGATTCGATAACGTGCTGATGGTG |
| q-GUS-R | TCAATCACCACGATGCCATGTTCATC |
| AtActin-F | GGTAACATTGTGCTCAGTGGTGG |
| AtActin-R | GCAGCATGAAGATTAAGGTCGTT |
