# Supplementary material for: Molecular Cloning and Functional Identification of a Pericarp- and Testa-Abundant Gene’s (AhN8DT-2) Promoter from Arachis hypogaea
Source: Int J Mol Sci. 2024 Jul 12;25(14):7671. doi: 10.3390/ijms25147671 (PMC11276643; doi:10.3390/ijms25147671)
Supplement: Supplementary file 1 [file ijms-25-07671-s001.zip › Table S6.pdf]

Table S6. Transcriptome expression of the *AhN8DT-2* gene in response to different hormones and ddH$_2$O.

Condition	FPKM
Leaf treated with abscisic acid	0
Leaf treated with brassinolide	0
Leaf treated with ddH2O	0
Leaf treated with ethephon	0
Leaf treated with paclobutrazol	0
Leaf treated with salicylic acid	0
