# Supplementary material for: Molecular Cloning and Functional Identification of a Pericarp- and Testa-Abundant Gene’s (AhN8DT-2) Promoter from Arachis hypogaea
Source: Int J Mol Sci. 2024 Jul 12;25(14):7671. doi: 10.3390/ijms25147671 (PMC11276643; doi:10.3390/ijms25147671)
Supplement: Supplementary file 1 [file ijms-25-07671-s001.zip › File S1.pdf]

File S1: CDS, protein, and Promoter sequences of *AhN8DT-2* gene

CDS sequence

>AH11G07230.1

ATGGCTTCCACTTCCAAGCTTCTTCTTGGCTCATCATTATTTCCTCATCCTACT
CCATCATCTCTTTCCATCACAACTGGTTCAATTGCAAGAAGAGCTTTATGGCA
CAAGAAGAGAAAACTCCAATCCTTGGAGCATAACTGGAACCATTCTTACAAA
TTCATTCATGGAGGATCCACACCTCATAAACCCAAGACACGAAATTTTCTTAT
GAAAGCAACTTCTGAACCGTTTGATACACAAACCATTTGGAAGTCTACCAAA
GATATCTTGCATACTGTCCAAAAGTTTACTATATCTTATTATTTAATTACTGTG
TTAATGAGCATATCTTCGACATCACTCCTTGCAGTGGAAAATTTATCAGACAT
ATCTCCAAAGTTTTTTGTTGGTTTATTCAAGTTTATCATAGCTATGATCTTCAT
GCATTGGTATACAGTTGGCATAAATCAATTAGCTGATCTTCAAATAGATAAG
ATTAACAAGCCATATCGTCCTTTGGCATCTGGAAAGATTTCCTACAAAGCTGG
AGTCATTATCACTATATCAAGTTTATTTATGAGTTTTGGACTTGGGTGGATAA
CAAAATCAAAGCCATTACTGTGGTGTCTCATTATATATTTTGTGGCAAACACA
AGTTATTCAATTAATTTGCCACTGTTAAGGTGGAAGAAATCTACAATTTCTAC
ACTATTGTCAGGTGTACCAACTATGCTAATAGCAAATAATGTTGCACCTTTTC
TTCATATGAAGACTTATGTTCTCGAGAAGACAACTATATTTCCAAGATCAATA
GTCTTTACCATTTTGGTCATGAGCTTTTACTATTCAGTCATCATATTGTCTAAG
GACATACCCGACATTGAAGGAGACAAGGCAGCCGGTATACAAACTTTGTCAG
TACGCTTAGGTCCAAAGCTGGTATTCTGGACATGTGTTTTACTCCTTGAAATG
GCTTATGGAATTGCCATTATCATGGGAGCAATATCTCCCTTCTTATGGAGTAA
AATTTTTACGGTCTTAGCACATGGAATCATGGCTGTGATTTTATGGTATCGTG
CTAATTCTGTTGACTTAAAGAGCAAAGATGCCTCCCAATCCTTTTATATGTTT
ATCTATAAGCTTCTTTGTGTGGAGAACATCCTTATACATTTCGTGAGATGA

Protein sequence

>AH11G07230.1

MASTSKLLLGSSLFPHPTPSSLSITTGSIARRALWHKKRKLQSLEHNWNHSYKFIH
GGSTPHKPKTRNFLMKATSEPFDTQTIWKSTKDILHTVQKFTISYYLITVLMSISST
SLLAVENLSDISPKFFVGLFKFIIAMIFMHWYTVGINQLADLQIDKINKPYRPLASG
KISYKAGVIITISSLFMSFGLGWITKSKPLLWCLIIYFVANTSYSINLPLLRWKKSTI
STLLSGVPTMLIANNVAPFLHMKTYVLEKTTIFPRSIVFTILVMSFYYSVIILSKDIP
DIEGDKAAGIQTLSVRLGPKLVFWTCVLLLEMAYGIAIIMGAISPFLWSKIFTVLA
HGIMAVILWYRANSVDLKSKDASQSFYMFIYKLLCVENILIHFVR

Promoter sequence

>AH11G07230

AGTAAGTCGTGATCATCACTATTGAATAGGGTTACAACTTTGAGTACAAAAC
TTTTTTTATAATATAAATAACAGAATTTATAATTCCGAGATATTATCTAATGT
GTAATAACTCTCAGTTTTGTTGTTAATTTAGAGTATAAAACGTAAGTCTTAGA
TTAGAAACAAATACTTTAAATCTTTTCTAAAGTAAGAACAGTTTTTTGAGTTA
GAGAAAGAACATTGTCCCAACTAGCAAAGAGTAGTTTAAATTAAATACTTCC
TTTGTCCAAAATTTTGAACTTCTTCATAATTTTGTAATATCTACGTTGCATGTA
TCTATTTTCTACTTCCTTCTTTTATTTTTCTTGATGTGTCTTTATACCACAAATT
TGTAAAATTAACTTTATAATCTCACATAGAGTTGGTACCACTTATTTGTCACA
GATGAGAGAGACTTCTTTTATGATGATAATAAATAAGTTGAGCAAATTTGTTT
TTCGTAGACAAAATATTAACATTTGAGATATTTTTTTAAGAGAAAGTTAAATT
AAAATATGTTTCTAATTTTTCAGAATTACGTGATGATTACTTTTAGTTTAAAAT
TTTTTATGTTTTAAACTTTACAATTTACACTTAAGTAATTAATTATAAGTCATA
ATAAAATTTTTAAATTAAATAAATCTCATTATATTCACAATTTTAAAAGGCAT
TTGAATCTAATAATAAATAGACGAAGAAAAAAGTAATAAAGAATACTTTATA
ATATTGGATGAATATATATCTTTTATATGATAAAAAAAACACAAAATTAACT
ATAGTATAATCTCAAAGCATCATATTCGATTCTTAAAAACATTGATTATTTAT
AAAAGACAATTCACATTAATGCTAGGAAATCAACATTATAGTATCATTTAATT
TTAACCAATACTATTTTAGGTTGCTAACAAACTCAGAATGAAATCTATTAAAA
AGGAAGTTTTTGTTGATTTTGAATTTTAAATTTTTTTAAGTTGAATACTTCTAT
GAAAATATCTTCATGTAAAAGTTGTATTGTGAATAATTAGATATTCATATATT
TTGACTCAATATGTTTAACTAAATTATCTAACGATTTATAATACTATTTTTATA
TGAAATCATCTTCATGAAATTAAAGTAGCCATTTTTTTAATGAATTTTAAATA
TTCTTATTTTTAATAGTTTTAAGTTTGTTTTTGCGTTAAATATTGGCTACTATA
ATATTAGTTGTATAATGTGGATAATATATATATAGTTTAATTATTCTCTTAGTC
ACTATAATTTTATCAAATATGTAATTAGATTTTTTATATTTTTTTATTGGATCT
CTACATTATTTTTAATTTTATAATTAAGTCTTTTTTATATAAAAAATATTAAAA
TTAATAGAATAATATTTCTCTCAAAAATATATAGTCAAAAATCTAATTAAATT
TTTAATTATGAATATTTGCAGAAAAATATTCTATTAACTCTAAGATTTTTTATG
GTATAAGAATTCAAATTAAAAAAAAAAAGTAGTGATAGGAATTCAAATAAAA
AAAATATAAAAATCTAATTATAAATTTAGTAAAATTATAGAAACTAATAATA
TATATATATATATATATATATATATATATATATAGAATTCTGCCGAATCCGGTTA
TTATTATTTGTAATTGTAACTAAATCATACTTTCATTGTTATGCTTTGCACATA
ATTGAAAAATTATATAAGTAGCTATATATATCCCCTTCTTAATAAACAGGACA
AGTGAAGATCTTGTCAAGAACACACAGTTTTCATGTCTTGATTGATTGTGATC
CAAAATTTGTTGATTCCCA***ATG***GCTTCCACTTCC
